# Haemoglobin levels for population from Gambo, a rural area of Ethiopia, and their association with anaemia and malaria

**DOI:** 10.1186/1475-2875-12-435

**Published:** 2013-12-01

**Authors:** Maria A Santana-Morales, Maria A Quispe-Ricalde, Raquel N Afonso-Lehmann, Pedro Berzosa, Jacob Lorenzo-Morales, Gabriel Tiziano, Francisco Reyes, Agustin Benito, Basilio Valladares, Enrique Martinez-Carretero

**Affiliations:** 1 University Institute of Tropical Diseases and Public Health of the Canary Islands, University of La Laguna, Astrofisico Francisco Sanchez s/n, 38207 Tenerife, Spain; 2 Gambo General Rural Hospital, Box 121, Shashemane, Ethiopia; 3 National Centre of Tropical Medicine, Institute of Health Carlos III, Monforte de Lemos n5, pabellón 13, 28019 Madrid, Spain

**Keywords:** Haemoglobin, Anaemia, Malaria, Ethiopia

## Abstract

**Background:**

Knowledge of appropriate reference intervals is critical not only to provide optimal clinical care, but also to enrol populations in medical research. The aim of this study was to generate normal ranges of laboratory values for haemoglobin among healthy Ethiopian adults and children and to determine if anaemia is a possible indicator of malaria in women and children in this area of Ethiopia.

**Methods:**

This study was carried out from January 2008 to May 2010. The reference sample population with malaria-negative consisted of 454 individuals, divided women, men and children. The malaria-infected sample population consisted of 117 individuals. The reference ranges were based on the guidelines from the Clinical and Laboratory Standards Institute. Haemoglobin concentration was determined by Hemo-Control EKF Diagnostic Analyser on whole blood. Testing for malaria-positive and negative infection was done by microscopy and by PCR.

**Results:**

The lower limits for adult haemoglobin range obtained from this population were slightly higher than those derived from other African populations, but were equal to those established by other studies in Ethiopia and the World Health Organization (WHO). Regarding children, the minimum values were lower than those obtained from different African populations and those established by WHO. The malaria-negative group had anaemia in 35.6% of cases and in the malaria-positive group in 70.9%. There was a stronger, statistically significant association between anaemia and malaria-positive samples than between anaemia and malaria-negative samples in women and both groups of children.

**Conclusions:**

The results from this study are a contribution in the definition of the haemoglobin parameters in African populations, which could be taken as standards for interpretation of laboratory results. The haemoglobin indices in adults from Gambo tended to be higher than other African populations and in children were lower than other studies in Africa. The results also suggest that anaemia is not useful as a supportive diagnostic criterion to monitor and evaluate malaria in women and children from Ethiopia, because a 29.1% of malaria cases will be not detected, because of not having anaemia.

## Background

Locally determined reference values are of immense clinical importance when taking into account intra- and inter-population variations, so that the correct clinical interpretation of laboratory results can be made [1]. The reference intervals of haematological indices currently used in Africa are derived from data collected from Europe or North America with predominantly Caucasian populations [2-4]. Even within the African continent, it is likely that substantial differences exist because of vast diverse climate, geographic altitude, co-infections, ethnic origin and human genetics, all of which can influence laboratory reference range parameters within a population [5-10]. This emphasizes the importance of population-specific reference values for different population settings [6,11]. Therefore, it is unlikely that any one set of reference haematological values can be applied across the globe for the same purpose. For example, African populations are reported to have lower haemoglobin than their western counterparts [5,12,13]. Consequently, using reference intervals determined from Caucasian populations in clinical trials among African populations might lead to the mistaken interpretation of ‘abnormal’ values in these populations [5,8,14].

Reference values for various blood parameters can be derived from healthy volunteers, from subjects attending health screening clinics or having annual medical examinations [15]. Such parameters are also important for measuring disease progression and response to therapy [5,8,14]. Knowledge of appropriate reference intervals is critical not only to provide optimal clinical care, but also to enrol populations in medical research. This is particularly relevant for African countries where there is emergence and re-emergence of some infectious diseases, whose management and prognosis heavily depend on laboratory values. It is also important for research and the evaluation of clinical trial-associated toxicity and adverse events of novel interventions against tuberculosis, malaria and infection with human immunodeficiency virus (HIV) [14].

Clinical diagnosis is widely used for the diagnosis of malaria, especially in resource-poor countries. Haematological changes in malaria, such as anaemia, thrombocytopaenia and leucocytosis or leucopaenia are well recognized [16]. The World Health Organization (WHO) and Roll Back Malaria (RBM) partnership have recommended that anaemia should be used as an additional indicator to monitor the malaria burden at community level and interventions on a national scale [17]. To establish anaemia as a malaria indicator, it is necessary to know haemoglobin values in each population.

Anaemia is an important contributor to malaria-attributable deaths in hospitals, with severe anaemia accounting for between 17 and 54% of malaria-attributed deaths in children under five years of age [18-20]. Therefore, the prevalence of (symptomatic and asymptomatic) anaemia in the community, as measured in population-based surveys, is used as the candidate indicator of the total malaria-related disease burden [21].

The aim of this study was to generate normal ranges of laboratory values for haemoglobin in Gambo village. To obtain the results of this study were assessed the haemoglobin parameter in healthy adults and children, and contrasted with that previously reported from African and Caucasian populations. In addition, this study suggests anaemia as a possible indicator of malaria in women and children in this area of Ethiopia as recommended by WHO and RBM.

## Methods

### Subjects

All haemoglobin values were taken at the Gambo General Rural Hospital, from patients attending health screening. Gambo is 2,200 m above sea level and lies 245 km southeast of Addis Ababa. The prevalence of malaria for the country is 8% [22]. The reference sample population with malaria-negative consisted of 454 individuals, made up of 127 women and 84 men between 13 and 98 years of age, 126 children between one and five years of age and 117 children between six and 12 years of age. The following categories were excluded: pregnant, malaria infected, HIV-positive and intestinal helminth-positive. The malaria-infected sample population, confirmed by microscopy, consisted of 117 individuals, 40 women and 14 men (13-98 years of age), 40 children (one to five years of age) and 23 children (six to 12 years of age), non-pregnant, HIV-negative and intestinal helminths-negative.

Patients were enrolled in this study from January 2008 to May 2010 at Gambo General Rural Hospital. Oral informed consent in local languages (Oromo and Amharic) was obtained. In the case of children, their parents or a head of household permitted the inclusion in the study. Clinical histories and samples were included in the study by code and the data were analysed anonymously. The research protocol had been reviewed and approved by the Ethical Committee of Gambo General Rural Hospital.

### Laboratory procedures

Haemoglobin concentration (g/dL) was determined by a Hemo-Control EKF Diagnostic analiser on whole blood. This analysis was performed within five min of collection. Testing for malaria-positive and negative infection was by microscopy and polymerase chain reaction (PCR). Haemoglobin concentration and microscopy was performed at the laboratory of the above-mentioned hospital and PCR were performed at the University Institute of Tropical Diseases and Public Health of the Canary Islands (IUETSPC). All experiments were performed according to good laboratory practice using appropriate standard operating procedures by well-trained laboratory staff.

### Microscopy and PCR testing

Microscopy for detection of *Plasmodium* species was performed using thick and thin blood smears [23], with two independent readings and a third reading for slides with discordant results. Malaria-positive infection was defined as the presence of any asexual malaria parasites detected on a thick peripheral blood smear. Finger-prick samples on 3MM filter paper were obtained for the PCR assays. Each filter paper specimen was stored in a plastic bag at room temperature and shipped to the IUETSPC. DNA extraction was performed using commercial kits (Speedtools tissue DNA Extraction Kit, Biotools, Madrid, Spain) and malaria-negative samples and *Plasmodium* species were confirmed by microscopy and nested PCR targeting the small subunit ribosomal RNA gene [24].

### Statistical analysis

Statistical analysis was based on the guidelines of the Clinical and Laboratory Standards Institute (CLSI: formerly National Council of Clinical Laboratory Services). Median and 95% reference ranges were determined for haemoglobin parameters and analysed by age and gender for adults. All calculations for determining reference ranges were based on the guidelines found in CLSI, document C28-A2 [1]. The normal distribution was tested by the Kolmogorov-Smirnov test. The sample population was grouped according to the age and gender distribution, and the median was used as a measure of central tendency. To eliminate bias due to small sample size, a bootstrap analysis method was used as a robust method to assess the 95% reference ranges from the Kolmogorov-Smirnov test. For each parameter, 10,000 bootstrap samples were selected [25] and the lower 95% reference limit was defined as the 2.5 percentile, while the upper limit was defined at the 97.5 percentile. Mann-Whitney U test was used to test for differences of means. Tests for an association between malaria and haemoglobin values were carried out by means of bivariate analysis: categorical variables were created for each group. The performance of the haemoglobin values was compared to those malaria-positive samples, in terms of their positive and negative predictive values (PPV and NPV).

An error probability (*P* value) of <0.05 was considered significant. All statistical calculations were performed with SPSS19 statistical and Resampling Stats for Microsoft Excel 2007 software.

## Results

A total of 454 malaria-negative individuals participated in this study. Men were marginally older than women, with mean ages of 40 and 32 years old, respectively. The mean age of child participants at study entry was two years old (one to five years of age) and nine years old (six to 12 years of age).

**Figure 1:**
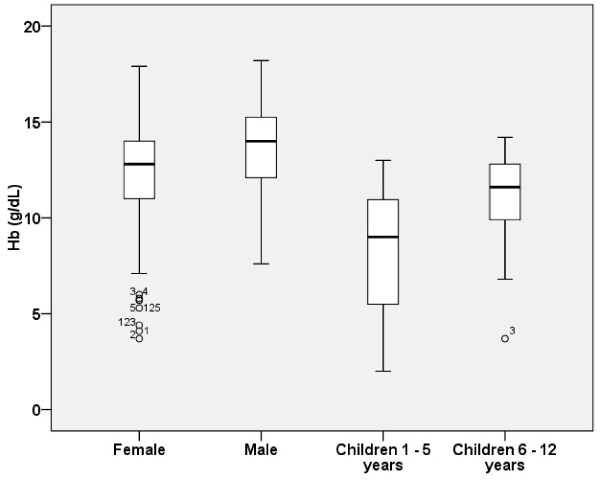
**Box and whisker plots showing variation in haemoglobin (Hb) values in malaria-negative Ethiopian adults and children.** n = 127 for female samples, n = 84 for male samples, n = 126 for child samples between one and five years old, n = 117 for child samples between six and 12 years old.

All haematological values were normally distributed. The analysis of haemoglobin values of these patients showed a mean, median and 95% reference range values stratified by age and gender, which are shown in Table 1. The lower haemoglobin value in non-malaria infected was 11.8 g/dL, 12.9 g/dL, 8.6 g/dL, and 9.7 g/dL in women, men and children between one and five years old and children between six and 12 years old, respectively. Women presented lower haemoglobin values than men (mean 12.2 *vs* 13.5 g/dL p < 0.05). Box and whisker plots for haemoglobin values by gender and age are shown in Figure 1.

**Table 1:** Mean, median and 95th percentile reference ranges of haemoglobin level for 454 Ethiopian malaria-negative adults and children

	**Subject group**			
	**Female (n = 127)**	**Male (n = 84)**	**Children 1-5 years (n = 126)**	**Children 6-12 years (n = 117)**
**Haemoglobin (g/dL)**				
**Mean ± SD**	12.2 ± 2.7	13.5 ± 2.4	9.1 ± 3.0	10.3 ± 3.1
**Median**	12.8	14 (<0.05)*	9.5	10.5
**95% range**	11.8-12.7	12.9-14.0	8.6-9.6	9.7-10.8

*P value (Mann-Whitney U test) for comparison of medians for adult subjects.

The means and 95th reference intervals for the Gambo General Rural Hospital population evaluated in the present study were compared with parameters obtained from other African populations and with parameters set by WHO. In general, the lower limits for adult haemoglobin range obtained from this population were slightly higher than those derived from Kenya [12], Uganda [5] and Gambia [14], but were equal to those established by another study in Ethiopia [26] and the haemoglobin range established by WHO (2011) [27]. In the child populations, the minimum values were lower than those obtained from different African populations and those established by WHO (Table 2).

**Table 2:** Comparison of haemoglobin values

	**Haemoglobin values by country and reference**						
**Subjects**	**Present study**	**Ethiopia **[26]	**Kenya **[12]	**Uganda **[5]	**Gambia **[14]	**Tanzania **[28]	**WHO **[27]
**Female**	11.8-12.7 (12.2)	12.2-16.6 (14.3)	5.9-10.0 (8.4)	9.9-13.7 (12.4)	9.8-15.0	NA	12.0-15.0
**Male**	12.9-14.0 (13.5)	13.9-18.3 (16.1)	8.3-11.3 (9.9)	11.5-17.1 (14.4)	11.1-16.6	NA	13.0-17.0
**Children 1-5 years**	8.6-9.6 (9.1)	NA	NA	8.8-12.5 (10.8)	8.7-13.4	8.1-13.9 (11.3)	11.0-14.0
**Children 6-12 years**	9.7-10.8 (10.3)	NA	NA	10.0-13.7 (11.8)	9.5-14.4	10.3-14.7 (12.6)	11.5-15.5

Values are the 95th reference intervals and in brackets are means.

WHO data are from above sea level.

NA: not available.

In the 117 malaria-positive samples, asexual malaria parasites were observed in the blood smears. The mean of haemoglobin values for the malaria-positive individuals group was determined and stratified by sex and age (Table 3). Of the malaria-positive slides, 51.3% (n = 60) were *Plasmodium vivax,* 40.2% (n = 47) were *Plasmodium falciparum* and 8.5% (n = 10) were mixed (*P. vivax* and *P. falciparum*).

**Table 3:** **Mean, median and 95**th **percentile reference ranges of haemoglobin levels for 117 malaria-positive adult and children from Gambo (Ethiopia)**

	**Subject group**			
	**Female (n = 40)**	**Male (n = 14)**	**Children 1-5 years (n = 40)**	**Children 6-12 years (n = 23)**
**Haemoglobin (g/dL)**				
**Mean ± SD**	9.3 ± 3.4	10.4 ± 2.7	6.4 ± 2.5	7.7 ± 3.2
**Median**	10.2	10.3	6.6	8.2
**95%range**	8.3-10.5	8.8-11.9	5.6-7.2	6.4-9.1

Haemoglobin values of the malaria-positive individuals were compared with those of the sex and age-specific malaria-negative groups using Mann-Whitney U test. For the different groups, the mean values of haemoglobin were significantly (*P* < 0.05) lower for infected groups than for the uninfected groups among women, men and children.

According to the results of the study, new anaemia values are defined, in the Gambo population, as a haemoglobin level under 11.8 g/dL, 12.9 g/dL, 8.6 g/dL, and 9.7 g/dL in women, men and in both groups of children, respectively. The malaria-negative group had anaemia in 35.6% of cases and the malaria-positive group in 70.9%. There was a stronger, statistically significant association between anaemia and malaria-positive samples than between anaemia and malaria-negative samples in women, and both groups of children (Table 4). The PPV of three age groups (women, children between one to five years of age, and children between six to 12 years of age) were 38%, 38.6% and 25%, respectively. In the cases of NPV were 85%, 90% and 90% respectively.

**Table 4:** **Adjusted odds ratios (OR) and 95**% **confidence intervals (CI) for haemoglobin values associated with malaria infection**

	**Malaria-positive**		
	**Female**	**Children 1-5 years**	**Children 6-12 years**
**OR**	3.5	5.8	2.9
**95% CI**	(1.7, 7.3)	(2.5, 13.7)	(1.1,7.3)
***P-*** **value**	0.001	0.001	0.022

If the malaria-positive group with anaemia is analysed, it observed in women that 45% infected by *P. vivax* and 78% by *P. falciparum* showed anaemia. In children, between one to five years of age, 74% infected by *P. vivax,* and 82.3% by *P. falciparum* showed anaemia and in children, between six to 12 years of age, showed anaemia in 54% infected by *P. vivax,* and 85.7% by *P. falciparum*. In women and both groups of children infected by different *Plasmodium* species, their mean values of haemoglobin were not significantly different (p > 0.05). In men, the haemoglobin value mean in malaria produced by *P. falciparum,* or *P. vivax* was not calculated due to the low number of malaria-positive samples.

## Discussion

Clinical laboratory values provide important data to help assess the health of an individual. In the absence of locally derived reference values for African populations, clinicians and researchers have had to use reference values from Caucasian populations. Differences in haematological values among distinct populations have been known for a long time and are discussed by several authors. Previous studies have shown that such values vary with age, ethnic origin, sociodemographic characteristics, genetic differences and environmental context [5,9,12,29]. The use of only Caucasian reference ranges for clinical management could lead to unnecessary treatment. The development of region- and age-specific reference values is thus essential for efficient patient management and the proper performance of clinical research.

WHO has designated haemoglobin, among other values, as essential to be measured by laboratory services in sub-Saharan Africa [30]. This study is the first description of haemoglobin ranges in the Gambo General Rural Hospital (Ethiopia). This haemoglobin range may serve in this area as standard for interpretation of laboratory results. This study has not only allowed the definition of haemoglobin ranges for a rural area in Ethiopia, but also provided evidence that reference intervals from one population should not be applied universally [31].

The haemoglobin indices in women and men from Gambo tended to be similar to Caucasian population intervals or higher than other African populations. This finding is consistent with previous studies in healthy and HIV-uninfected populations in other Ethiopian regions [12,32]. These findings could be attributable to the geographic location of Gambo area (altitude 2,200 m above sea level). This speculation is corroborated by findings from other studies that reported higher haemoglobin levels among people living at high altitudes [32]. Altitude-induced erythropoiesis and/or dietary factors could play a role in causing these variations [12].

However, haemoglobin values for children between one and five years of age (8.6-9.6 g/dL) and children between six and 12 years of age (9.7-10.8 g/dL) were lower than those reported from other studies in Africa and among Caucasian populations (see Table 2). Surprisingly, the 2.5th and 97.5th percentile for haemoglobin ranges for Gambo children is lower than standard in industrialized and African countries. This wider range may be attributable to several factors, including poor nutritional status resulting in iron deficiency, genetic disorders (eg, thalassemia, sickle cell trait) [9], which was not checked in the population included in this study, or an important deficit of haemoglobin from living at over 2,000 m above sea level. There was no evidence of a difference between boys and girls in the haemoglobin parameter, which is consistent with previous studies, showing that differences between genders become evident only at about 12 years of age [33,34].

Following recommendations of the CLSI guidelines for laboratory indicators and Canadian laboratory initiative on paediatric reference intervals [1,35], a robust bootstrap analysis was used to eliminate bias. In addition, this study included only ambulatory subjects who were otherwise apparently healthy, their individual health status was assessed for the same subclinical condition as HIV, intestinal helminths, pregnancy, and malaria, known to interfere with the obtained parameters. In the context of resource-limited settings, the methods included in this study were sufficient to determine reference ranges for use in this population.

This study presents the first description of haemoglobin ranges for adults and children in the Gambo General Rural Hospital (Ethiopia). The development of this reference range may provide guidelines to be used by local health practitioners in patient management within this region and for the design, performance and evaluation of clinical research. The findings obtained in this study were comparable to those of other studies within Africa [5,12,14,26-28]. Thus, they could be used as reference ranges for adults and children within the Gambo General Rural Hospital that presents a radius of action 100 km. These haemoglobin ranges provide anaemia values, which can be used to monitor the malaria burden at the population level, and that use Gambo General Rural Hospital as a reference hospital, as recommended by WHO and RBM.

Finally, WHO defines anaemia as a haemoglobin concentration of <12.0 g/dL, <13.0 g/dL in females and males, and 11.0 g/dL and 11.5 g/dL in children between one and five years old and those between six and 12, respectively [36]. Anaemia was defined in this study as haemoglobin values of <11.8 g/dL, <12.9 g/dL in women and men, which are similar to WHO. In children, anaemia was defined as haemoglobin values of <8.6 g/dL and <9.7 g/dL for the two groups mentioned above, respectively. Child values are lower than WHO values, as explained above. Malaria-negative groups presented anaemia in 35.6% of cases of all analysed sample. Malaria-positive groups did not have anaemia in 29.1% of cases of all analysed sample. The anaemia in malaria-positive groups was presented in 70.9% of the cases: this anaemia could be divided into 35.6% of anaemia cases caused by unknown reason, as occurred in the malaria-negative groups, and into 35.3% of anaemia attributed to malaria. Hence, the presence of malaria doubles the presence of anaemia. Malaria can produce the same percentage of anaemia as other pathologies that cause anaemia. Women and both groups of children with malaria were 3.5, 5.8 and 2.9 times, respectively, more likely to have anaemia than women and children with malaria-negative data. This association between anaemia and malaria in women and children is consistent with previous reports, which indicate that anaemia values is a sensitive indicator to scaling up malaria interventions and may thus be used as a proxy indicator to track the burden of malaria [37]. Previous articles indicate that anaemia in Ethiopia is mainly due to malaria [38,39]. The PPV of 38%, 38.6% and 25% in each groups, could be in accordance with a malaria low prevalence in study area, nevertheless, despite of the NPV of 85%, 90% and 90% obtained here, a negative anaemia result does not rule out malaria-positive [40]. Based on these results, anaemia values may have limited utility in this area as indicator of malaria infection.

## Conclusions

The results from this study are a contribution in the definition of haemoglobin parameters in African populations, which could be taken as standard for interpretation of laboratory results. The haemoglobin indices in adults from Gambo tended to be higher than in other African populations and in children were lower than other studies in Africa.

The results also suggest that anaemia is not useful as a supportive diagnostic criterion to monitor and evaluate malaria in women and children from Ethiopia, because 29.1% of malaria cases will be not detected, because of not having anaemia.

## Competing interests

The authors declare that they have no competing interests.

## Authors’ contributions

MASM carried out the sample collection in Gambo (Ethiopia), the microscopy and molecular study, the analysis and interpretation of data and prepared the manuscript. MAQ, RNAL and JLM helped with the statistical analysis and interpretation. PB and AB helped with the performance of molecular studies. FR and GT helped with the collection of samples and analysed haemoglobin values. BV helped to draft the manuscript and EMC coordinated and funded the study and drafted the manuscript. All authors read and approved the final manuscript.

## References

[B1] National Committee for Clinical laboratory Standards (NCCLS) How to define and determine reference intervals in the clinical laboratory NCCLS C28-A2. Vol. 20. (13) 2000 2 Wayne PA USA: National Committee for Clinical and Laboratory Standards

[B2] Beutler E Lichtman M Coller B Kipps T Selighohn U Williams Hematology 2001 Columbus, OH, USA: McGraw-Hill

[B3] Lewis S Dacie and Lewis’s Practical Haematology 2001 9 New York, NY, USA: Churchill Livingstone

[B4] Wintrobe M Lee G Wintrobe’s Clinical Haematology 1999 10 Baltimore, USA: The Williams & Wilkins Co.

[B5] Lugada ES Mermin J Kaharuza F Ulvestad E Were W Langeland N Asjo B Malamba S Downing R Population-based hematologic and immunologic reference values for a healthy Ugandan population Clin Diagn Lab Immunol 2004 11 29 34 14715541 10.1128/CDLI.11.1.29-34.2004 PMC321349

[B6] Ernst E Schrader M Saradeth T Bergmann H Analytical and physiological variations of some haemorheological and haematological bloods tests Clim Haemortheol 1990 10 Suppl 5 525 533

[B7] Mangwendeza MP Mandisodza A Siziya S Haematology reference values for healthy elderly blacks residing in Harare, Zimbabwe Cent Afr J Med 2000 46 Suppl 5 120 123 11210332 10.4314/cajm.v46i5.8534

[B8] Quintó L Aponte J Sacarlal J Espasa M Aide P Mandomando I Guinovart C Macete E Navia MM Thompson R Menendez C Alonso PL Haematological and biochemical indices in young African children: in search of reference intervals Trop Med Int Health 2006 11 1741 1748 10.1111/j.1365-3156.2006.01764.x 17054755

[B9] Karita E Ketter N Price M Kayitenkore K Kaleebu P Nanvubya A Anzala O Jaoko W Mutua G Ruzagira E Mulenga J Sanders EJ Mwangome M Allen S Bwanika A Bahemuka U Awuondo K Omosa G Farah B Amornkul P Birungi J Yates S Stoll-Johnson L Gilmour J Stevens G Shutes E Manigart O Hughes P Dally L Scott J Stevens W CLSI-derived hematology and biochemistry reference intervals for healthy adults in eastern and southern Africa PLoS One 2009 4 e4401 10.1371/journal.pone.0004401 19197365 PMC2632744

[B10] Bain B Ethnic and sex differences in the total and differential white cell count and platelet count J Clin Pathol 1996 49 664 666 10.1136/jcp.49.8.664 8881919 PMC500612

[B11] Dapper DV Nwauche CA Didia BC Haematological reference values for healthybadults in Port Harcourt, Nigeria Port Harcourt Med J 2006 1 25 28

[B12] Kibaya RS Bautista CT Sawe FK Shaffer DN Sateren WB Scott PT Michael NL Robb ML Birx DL de Souza MS Reference ranges for the clinical laboratory derived from a rural population in Kericho, Kenya PLoS One 2008 3 e3327 10.1371/journal.pone.0003327 18833329 PMC2553265

[B13] Tugume SB Piwowar EM Lutalo T Mugyenyi PN Grant RM Mangeni FW Pattishall K Katongole-Mbidde E Hematological reference ranges among healthy Ugandans Clin Diagn Lab Immunol 1995 2 233 235 7697535 10.1128/cdli.2.2.233-235.1995 PMC170134

[B14] Adetifa IMO Hill PC Jeffries DJ Jackson-Sillah D Ibanga HB Bah G Donkor S Corrah T Adegbol RA Haematological values from a Gambian cohort – possible reference range for a West African population Int J Lab Hematol 2009 31 615 622 10.1111/j.1751-553X.2008.01087.x 18631172

[B15] Bain B Normal Range in Blood Cells A Practical Guide 2006 4 New Jersey: Wiley-Blackwell 199 216

[B16] Erhart LM Yingyuen K Chuanak N Buathong N Laoboonchai A Miller RS Meshnick SR Gasser RA Jr Wongsrichanalai C Hematological and clinical indices of malaria in a semi-immune population of Western Thailand Am J Trop Med Hyg 2004 70 8 14 14971691

[B17] World Health Organization Minutes MERG Anaemia Task Force Meeting: 27-28th Oct 2003 http://whqlibdoc.who.int/publications/2005/9241593199_annex4_eng.pdf

[B18] Biemba G Dolmans D Thuma PE Weiss G Gordeuk VR Severe anaemia in Zambian children with Plasmodium falciparum malaria Trop Med Int Health 2000 5 9 16 10.1046/j.1365-3156.2000.00506.x 10672200

[B19] Marsh K Forster D Waruiru C Mwangi I Winstanley M Marsh V Newton C Winstanley P Warn P Peshu N Pasvol G Snow R Indicators of life-threatening malaria in African children N Engl J Med 1995 332 1399 1404 10.1056/NEJM199505253322102 7723795

[B20] Slutsker L Taylor TE Wirima JJ Steketee RW In-hospital morbidity and mortality due to malaria-associated severe anaemia in two areas of Malawi with different patterns of malaria infection Trans R Soc Trop Med Hyg 1994 88 548 551 10.1016/0035-9203(94)90157-0 7992335

[B21] Korenromp EL Armstrong-Schellenberg JR Williams BG Nahlen BL Snow RW Impact of malaria control on childhood anaemia in Africa – a quantitative review Trop Med Int Health 2004 9 Suppl 10 1050 1065 15482397 10.1111/j.1365-3156.2004.01317.x

[B22] Ayele DG Zewotir TT Mwambi HG Prevalence and risk factors of malaria in Ethiopia Malaria J 2012 11 195 10.1186/1475-2875-11-195 PMC3473321 22691364

[B23] John CC McHugh MM Moormann AM Sumba PO Ofulla AV Low prevalence of Plasmodium falciparum infection among asymptomatic individuals in a highland area of Kenya Trans R Soc Trop Med Hyg 2005 99 780 786 10.1016/j.trstmh.2005.04.012 16085173

[B24] Santana-Morales MA Afonso-Lehmann RN Quispe MA Reyes F Berzosa P Benito A Valladares B Martinez-Carretero E Microscopy and molecular biology for the diagnosis and evaluation of malaria in a hospital in a rural area of Ethiopia Malar J 2012 11 199 10.1186/1475-2875-11-199 22694993 PMC3489512

[B25] Efron B Better bootstrap confidence intervals J Am Stat Assoc 1987 82 171 200 10.1080/01621459.1987.10478410

[B26] Tsegaye A Messele T Tilahun T Hailu E Sahlu T Doorly R Fontanet AL Rinke De Wit TF Immunohematological reference ranges for adult Ethiopians Clin Diagn Lab Immunol 1999 6 410 414 10225845 10.1128/cdli.6.3.410-414.1999 PMC103732

[B27] World Health Organization Concentraciones de hemoglobina para diagnosticar la anemia y evaluar su gravedadhttp://www.who.int/vmnis/indicators/haemoglobin_es.pdf

[B28] Buchanan AM Muro FJ Gratz J Crump JA Musyoka AM Sichangi MW Morrissey AB M’rimberia JK Njau BN Msuya LJ Bartlett JA Cunningham CK Establishment of haematological and immunological reference values for healthy Tanzanian children in Kilimanjaro Region Trop Med Int Health 2010 15 1011 1021 20636301 10.1111/j.1365-3156.2010.02585.x PMC3024440

[B29] Saathoff E Schneider P Kleinfeldt V Geis S Haule D Maboko L Samky E de Souza M Robb M Hoelscher M Laboratory reference values for healthy adults from southern Tanzania Trop Med Int Health 2008 13 612 625 10.1111/j.1365-3156.2008.02047.x 18331386

[B30] Petti CA Polage CR Quinn TC Ronald AR Sande MA Laboratory medicine in Africa: a barrier to effective health care Clin Infect Dis 2006 42 377 382 10.1086/499363 16392084

[B31] Ezzelle J Rodriguez-Chavez IR Darden JM Stirewalt M Kunwar N Hitchcock R Walter T D’Souza MP Guidelines on good clinical laboratory practice: bridging operations between research and clinical research laboratories J Pharm Biomed Anal 2008 46 18 29 10.1016/j.jpba.2007.10.010 18037599 PMC2213906

[B32] Ezeilo GC Non-genetic neutropenia in Africans Lancet 1972 11 1003 1004 4116936 10.1016/s0140-6736(72)92409-9

[B33] Yip R Johnson C Dallman PR Age-related changes in laboratory values used in the diagnosis of anemia and iron deficiency Am J Clin Nutr 1984 39 427 436 6695842 10.1093/ajcn/39.3.427

[B34] Castro OL Haddy TB Rana SR Age- and sex-related blood cell values in healthy black Americans Public Health 1987 102 232 237 3104982 PMC1477821

[B35] Schnabl K Chan MK Gong Y Adeli K Closing the gaps in paediatric reference intervals: the CALIPER initiative Clin Biochem Rev 2008 29 89 96 19107221 PMC2605413

[B36] World Health Organization Control of Nutritional Anemia with Special Reference to Iron Deficiency Anemia 1975 Geneva: World Health Organization 812269

[B37] Mathanga DP Campbell CH Eng JV Wolkon A Bronzan RN Malenga GJ Ali D Desai M Comparison of anaemia and parasitaemia as indicators of malaria control in household and EPI-health facility surveys in Malawi Malar J 2010 9 107 10.1186/1475-2875-9-107 20409342 PMC2864286

[B38] Maina RN Walsh D Gaddy C Hongo G Waitumbi J Otieno L Jones D Ogutu BR Impact of Plasmodium falciparum infection on haematological parameters in children living in Western Kenya Malar J 2010 9 Suppl 3 4 10.1186/1475-2875-9-S3-S4 21144084 PMC3002140

[B39] Deribew A Alemseged F Tessema F Sena L Birhanu Z Zeynudin A Sudhakar M Abdo N Deribe K Biadgilign S Malaria and under-nutrition: a community based study among under-five children at risk of malaria, south-west Ethiopia PLoS One 2010 5 Suppl 5 e10775 20505829 10.1371/journal.pone.0010775 PMC2874013

[B40] Askling HH Bruneel F Burchard G Castelli F Chiodini PL Grobusch MP Lopez-Vélez R Paul M Petersen E Popescu C Ramharter M Schlagenhauf1 P Management of imported malaria in Europe Malar J 2012 11 328 10.1186/1475-2875-11-328 22985344 PMC3489857

